# Periscope of Dermatology

**Published:** 1894-08-11

**Authors:** 


### PERISCOPE OF DERMATOLOGY.

**Acne.**—Crocker[1] in a clinical lecture draws attention to the freedom of all hairy parts from acne rosacea and acne vulgaris, and he states that scars on such parts indicate acne varioliformis. With regard to treatment, he recommends salines by the mouth, and after removing the plugs with a watch-key, he applies a calamine lotion containing some mercuric-chloride in solution; later he gives a sulphur lotion, or an ointment of hypochlorite of lime, which must be freshly made. Kaposi[2] in describing some unusual forms of acne, gives the alternative name of folliculitis. His first case is one of acne varioliformis extending over the whole face and the front of the chest. Under the name of acne indurata he describes an eruption of hard bean-shaped papules occurring on the face, or on the extremities, accompanied with some itching. "Acne necroticans et exulcerans serpiginosa nasi" is the term applied to an eruption which occurs at the tip of the nose. It commences as a small nodule, the size of a pin's head, or of a pea, which soddens and undergoes necrosis; in this way the whole skin of the nose may be destroyed. In acne telangiectodes the eruption consists of granulation-tissue papules irregularly distributed over the whole body, but which do not necrose; these papules are the size of a pea; they have a succulent feel, and the surface is smooth, shiny, and pales under pressure; they are situated in the deeper layer of the corium about the hair follicules and sweat glands. Von Hebra and Ullman[3] recommend the following lotion for acne—icthyol 3i, sulphuric ether 3iii, alcohol 3vi ♏., fiat lotio. Leslie Roberts[4], treated persistent acne by electrolysis, using a current of the strength of 3 milliamperes. There is little or no risk of producing scars.

**Lupus.**—Fox[5] finds that out of 95 cases 48 per cent. began during the first ten years of life, and ten cases in the first year; in only four cases did the disease commence after the age of 40. There was evidence of glandular affection before the appearance of the lupus in over 30 per cent., associated scrofulous gummata in about 15 per cent., and in about 8 per cent. of the cases there had been bone or joint mischief. He mentions cases of true lupus arising from caseating glands, and from diseased bone or scrofulous gummata. Walker[6] in a paper read before the Edinburgh Medical Society, describes three varieties of lupus vulgaris: (1) destructive, (2) nodular, (3) fibrous; the latter mainly occurs on the limbs. He recommends free excision whenever possible. Schutz[7], while recognizing that excision combined with skin-grafting is the only rational method of treatment, considers that it is open to objection in cases of extensive ulcerating lupus of the face. In such cases he obtains favourable results by the following method: The patches are thoroughly scarified to the extent of one centimetre beyond the edge, and after the bleeding is stopped the surface is brushed over with a solution of chloride of zinc, made clear by the addition of hydrochloric acid. The pain, which is severe, and lasts about six hours, can be relieved by ice compresses. After this pyrogallic vaseline (1 in 4), is applied, and changed three times a day for four days; boric acid lotion is then applied for three days, when pyrogallic vaseline is re-applied for another four days. Healing is rapid. Brooke[8] comments on the above paper, but does not agree with the recommendation of excision, and advises the method of Veiel,[9], which consists in ploughing up the lupoid patch with a sharp stick of caustic potash, and then applying pyrogallol ointment. Harrison[10] recommends that the lupoid patch should be covered with a compress dipped in an 8 per cent. solution of sod. hyposulph. Next day this is removed, and hydro-chloride acid applied. This treatment is continued for eight days, and the patch cicatrizes. Sympson[11] recommends a solution of salicylic acid in collodion (ʒ1 to ℥1) for lupus; he does not propose to cure the disease, but to keep it in check. A daily application is necessary at first, but afterwards twice a week is sufficient. Stopford[12] urges the use of the thermo-cautery; he first removes crusts and scrapes the patch, then, with a fine pointed cautery he makes a succession of punctures a quarter of an inch deep close together all round the patch, the whole surface of which is then reduced to a leathery condition by the button cautery. Bramwell[13] records two cases treated by the internal administration of thyroid extract without any local treatment. He was led to try this on account of the fact that cases of myxœdema often die from tubercular affections; he does not profess to cure lupus by these means, but both his cases showed considerable improvement after some months treatment. Hutchinson[14], when showing a boy aged sixteen with twenty patches of the disease, stated that when lupus is multiple, the multiplicity is always at the outset; he also stated that there is no risk of visceral infection with tuberculosis from lupus. Hardaway[15] describes a case of tuberculosis of the skin simulating lupus erythematosus; it occurred in a man, aged twenty-eight, as a yellow nodule over the malar bone; this patch gradually spread over the nose in the same way as lupus erythematosus. Microscopic examination of a small piece showed tubercle bacilli, and for this reason the author does not believe the case was one of lupus erythematosus. Jamieson[16] describes a young woman, aged twenty, with a telangiectic variety of lupus erythematosus. It began when the patient was seven years old, and consists of a series of serpiginous bands on the face, chin, and scalp. Electrolysis was suggested.

**Pemphigus.**—Corlett[17], in an exhaustive paper upon pemphigus, describes two fatal cases of acute pemphigus foliaceous, and refers to others; he considers that the disease is contagious. A case of pemphigus, which proved fatal, is reported by Webber[18], death apparently being due to diarrhœa; no post-mortem was made. Another fatal case is reported by Penrose[19], and that, too, in a healthy woman, aged twenty-eight, who died of septicæmia within five weeks of showing symptoms of the disease. There was no visceral disease post-mortem. Another fatal case is reported by Robinson[20] where a child, aged nine months, developed gangrene of both legs and arms following an attack of pemphigus. Death occurred from collapse without any diarrhœa. Interesting cases of pemphigus are reported by Herman[21], and Cuthbert[22], both of which quickly recovered under simple treatment.

**Parasitic Diseases.**—Blaschko[23] calls attention to the spread of disease by barbers, the most common disease so spread is herpes tonsurans, but besides this, the following diseases can also be contracted in barber's shops: impetigo contagiosa, acne variolifomis, trichorrhœa nodosa, certain eczemas, alopecia areata, and syphilis. To prevent this danger the barber should wipe his razor with absolute alcohol, and scald the shaving brush after using; cotton wool should be used instead of a puff for powder. Jackson[24] contributes a paper on the same subject, and specially condemns the hair-clipper, stating that epidemics of alopecia in some French regiments have been traced to its use.

**Sycosis.**—Leistikow[25] recommends powders for slight cases, and a 2 p.c. solution of resorcin as a moist dressing when there is folliculitis; when the inflammatory symptoms are slight he prefers a paste of zinc-oxide, sulphur, talc, and lard. It can be coloured to resemble the skin by the addition of 1 p.c. of red sulphide of mercury. Jamieson[26] in a lecture on tinea barbæ, states that most cases are due to the trichophyton megalosporon. He considers that the disease may be transmitted from animals as well as by barbers. For treatment he orders super-fatted soaps, and sulphur in the form of an ointment. Bergonzini[27] has cultivated the fungus of trichomycosis nodosa, which he proposes to name monilia trichophilia. It was obtained from an affection of the upper lip. Biro,[28] after investigating the fungus of favus, finds that it behaves differently when cultivated in different media; after repeated inoculations the apparently different cultures lose their differential features, so it is probable that the fungus is really the same in all the cases. Gouladze[29] obtains rapid cure of favus by compresses saturated with the following lotion: ℞ Thymol, gr. 8; chloroform, ʒ 5; olive oil, ℥ 3. The diseased hair is removed by friction, and finally a solution of iodine in glycerine (2 in 1) is applied daily for a week. Tims[30] recommends the following ointment for tinea tonsurans: Acid-carbolic, acid-salicylic, a.a., ʒ js.; vaseline, ℥ 1. This is to be rubbed on night and morning. When this is not successful, he uses an ointment containing 10 to 20 grains of chrysorobin to the ounce.

**Alopecia.**—Abraham[31] regards a number of these cases as etiologically related to common ringworm. In 32 per cent. of his cases there was a previous history of tinea in the patient, or in other members of his family. He also quotes a series of cases occurring in an orphanage. For treatment he recommends the cautious use of Burt's fluid. Bowen[32] records an epidemic of alopecia in an asylum for orphan girls of from 3 to 14 years of age; 63 out of the 69 children were affected to a greater or less extent. There was no chance of infection from outside, but the disease was typical alopecia, and though there were no signs of ordinary tinea, yet it appeared to be contagious. Rogers[33] cites a case where there was no absolute bald patch, but only thinning of the hair of the whole head. Leistikow[34] finds a stick application more convenient than an ointment. He rubs the part thoroughly with a stick composed of chrysorobin 30 parts, cera flava 35 parts, colphonium 3 parts, olive oil 30 parts. Ferras[35] considers general treatment necessary, and orders strong sulphur baths for the whole body to be followed by massage. Locally, he gives iodine and a hot sulphur spray. Fox[36] comments on a paper by Dubreuilh on depilating folliculitis of the glabrous regions. The eruption is always situated upon the legs, knees, or lower parts of the thighs. There is a growth of granulation tissue about the hair-sheaths, but no micro-organism could be detected.

**Erythema.**—Finger[37] describes the microscopic examination of two cases of erythema multiforme; in both cases he found numerous mononucleated cells in the papillæ and in the sweat-glands; round these cells were found networks of cocci. The disease in the first case was complicated with diphtheria, in the second with purpura. He believes that the eruption is due, not to the absorption of toxic bodies, but to the metastatic deposit of the cocci themselves in the skin. This is the case in all erythemas which accompany suppurative processes. Comments on this paper are made by Bruck[38] and Adamson[39]. Widal and Therese[40] describe a case where streptococci gained entrance into the system by the tuberculous apex of the lung, causing profuse purpura and erythema. Patterson[41] describes three cases of erythema indurata of Bazin. In this disease an eruption of punched out ulcers resembling cutaneous syphilides appears on the front of the legs. It arises in scrofulous patients, and the author considers that it is not rare. It is quite distinct from scrofuloderma and lupus, and the disease can be easily distinguished from syphilis by its evident local character, and by its not yielding to anti-syphilitic treatment. Jamieson[42] also comments upon the same subject. Payne,[43] after referring to erythema occurring in the course of other diseases, or due to toxic absorption, describes several cases of persistent erythema, in which the affection remains fixed on certain parts of the skin for weeks, months, or years, without any alteration of type. The majority of his cases agree with the "erythema scarlatineforme" of French dermatologists. He does not enter into the etiology of the disease; but states that though the diseases has no tendency towards spontaneous recovery it can readily be cured by quinine or salicylate of soda. Smith[44] describes a case of erythema elevatum diutinum, for which he suggests the name of cutaneous fibromatosis. After excising a small nodule for microscopic examination, there was no recurrence, so he is inclined to urge removal of the tumours by the knife. Ramsey-Smith[45] describes a form of erythema allied to urticaria, which he considers is nervous in origin. He has seen cases at all ages, and it appears te have some epidemic characters. For chilblains, Pilatti[46] recommends digitalis both internally and externally as a lotion in combination with thymol and alcohol. He deprecates the use of ointments, &c., for this trouble.

**Eczema.**—Ravogli,[47] in a paper on the etiology of eczema, states that the presence of pus on the skin is one of the most common causes of the disease, and after experimental inoculation, assumes that eczema is developed by the growth of staphylococcus albus upon a previously-inflamed skin. He therefore considers that eczema is a local affection of the skin, which is contagious under favourable circumstances. Hutchinson[48] relates a case of red eczema in a child aged eleven years, which was apparently caused by hot weather. It had existed on and off since the age of one year. Schwimmer,[49] in a paper read before the International Medical Congress, maintains that the nerves have a predominating influence in the causation of the disease, and that the parasitic organisms are only accidental factors in certain forms. Herrkeimer[50] recommends the following lotion to be applied thrice daily in cases of eczema: ℞ peroxide of iron, gr. $\frac{3}{4}$; bismuth subnitrate zinc oxide a.a., gr. 15; glycerine, gr. $7\frac{1}{2}$; rose water, ℨ 9. M. Lassar[51] describes a form of eczema attacking surgeons' hands as the effect of antiseptics. He advises rubbing in a mixture of olive oil, glycerine, vaseline, and lanoline. Besnier[52] recommends the following ointment for impetigo: plaster of vigo, and vaseline, a.a. Blenheim[53] describes a fatal case of eczema in a child aged four months; the post-mortem changes pointed to acute septicæmia, and staphylococci pyogenes albus and aureus were found in the cerebro-spinal fluid, the blood, and the liver. He considers that these micro-organisms found their way into the blood by means of the lesions of the skin. He adds that in view of such bacterial invasion, it is advisable that a suitable antiseptic should be applied to the eczematous surface before any ointment is used.

1 Clin. Journ., Feb. 21, 1894, p. 269. 2 Arch. fur Derm. und Syph., 1894, No. 26, p. 87; Am. Med. and Sur. Bull., 1894, Mar. 1, p. 307. 3 Med. Week, March 9, 1894, p. 120. 4 Brit. Journ. of Derm., March, 1894, p. 150. 5 Ditto, p. 84. 6 Brit. Med. Journ., May 12, 1894, p. 1,024. 7 Arch. fur Derm. und Syph., No. 1, 1894; Centralblat fur Chir., No. 7, 1894, p. 155; Am. Journ. Med. Sci., April, 1894, p. 458. 8 Med. Chron., April 1894, p. 65. 9 Berl. Clin. Woch., No. 39; Ther. Gaz., Jan., 1894, p. 34. 10 Med. Rec., Jan. 20, 1894, p. 87. 11 Practitioner, Feb., 1894, p. 96; Ther. Gaz., May 15, 1894, p. 335. 12 Liverpool Med. Chir. Journ., No. 26, 1894; Ther. Gaz., March 15, 1894, p. 204. 13 Brit. Med. Journ., April 14, 1894, p. 786. 14 Clin. Journ., April 18, 1894, p. 397. 15 Am. Journ. Med. Sci., April, 1894, p. 408. 16 Edin. Med. Journ., March, 1894, p. 828. 17 Am. Journ., April, 1894, p. 413. 18 Lancet, Feb. 24, 1894, p. 472. 19 Ditto, Feb. 3, 1894, p. 265. 20 Ditto, March 24, 1894, p. 773. 21 Int. Med. Mag., Jan., 1894, p. 1,115. 22 Brit. Med. Journ., June 9, 1894, p. 1,237. 23 Berl. Klin. Woch., 1893; Am. Journ., March, 1894, p. 354. 24 Med. Rec., April 7, 1894, p. 426. 25 Med. Week, April 13, 1894, p. 179. 26 Clin. Journ., Jan. 24, 1894, p. 205. 27 Med. Rec., May 5, 1894, p. 566. 28 Arch. fur Derm., &c., 1894, No. 6. 29 Med. Week, May 4, 1894, p. 216. 30 The Hospital, April 21, 1894, p. 54. 31 Practitioner, Feb., 1894, p. 154. 32 B. Journ. of Derm., March, 1894, p. 80. 33 Brit. Med. Journ., April 28, 1894, p. 911; 34 Ep. Brit. Med. Journ., March 31, 1894, p. 361. 35 Ther. Gaz., Jan. 1894, p. 65. 36 B. Journ. Derm., April, 1894, p. 124. 37 Arch. fur Derm., &c., 1893, No. 5. 38 Fortsch. der Med., No. 7, 1894, p. 279. 39 B. Journ. of Derm., April, 1894, p. 123. 40 Med. Week, Feb. 10, 1894, p. 80. 41 Dublin Medical Journ., May, 1894, p. 439. 42 Ed. Med. Journ. March, 1894, p. 830. 43 B. Journ. of Derm., May, 1894, p. 129. 44 Ditto, ditto, p. 124. 45 Ed. Med. Journ., March, 1894, p. 800. 46 Ep. B.M.J., Feb. 24, 1894, p. 31. 47 Pract., Feb. 1894, p. 107. 48 Ditto, ditto, p. 123. 49 Lancet, April 14, 1894, p. 960. 50 Med. Week, Feb. 23, 1894, p. 95. 51 Ditto, May 4, 1894, p. 208. 52 Ther. Gaz., Feb. 1894, p. 126. 53 Central fur. Bakt., Feb. 5, 1894, and Ep. B.M.J., April 14.

(*To be continued.*)

